# Subspecialisation recognition in European Radiology—follow-up survey by the Accreditation Council in Imaging and European Society of Radiology National Societies Committee

**DOI:** 10.1186/s13244-025-02054-x

**Published:** 2025-08-15

**Authors:** Miraude Adriaensen, Paolo Ricci, Christian Loewe, Helmut Prosch, Mitja Rupreht, European Society of Radiology (ESR), on behalf of the European Board of Radiology (EBR)

**Affiliations:** 1 Department of Medical Imaging, Zuyderland Medical Center, Heerlen, The Netherlands; 2 Department of Radiological, Oncological and Pathological Sciences, Sapienza University of Rome, Rome, Italy; 3 Department of Biomedical Imaging and Image‑Guided Therapy, Medical University of Vienna, Vienna, Austria; 4 Radiology Department, UMC Maribor, Maribor, Slovenia; 5 Medical Faculty, University of Maribor, Maribor, Slovenia; 6 Faculty of Health Sciences, University of Primorska, Izola, Slovenia; 7 European Society of Radiology, Vienna, Austria

**Keywords:** Radiology subspecialisation, Diploma, Recognition, European Society of Radiology, National societies

## Abstract

**Objectives:**

To assess the status of radiology subspecialisation recognition across Europe, targeting European Society of Radiology (ESR) National Societies.

**Methods:**

A questionnaire was distributed to members of the ESR National Societies Committee regarding the recognition of radiology subspecialties in their respective countries.

**Results:**

Responses were received from 37 out of 47 countries (78%). Radiology subspecialties are recognised in 25 countries, whereas in 12 countries, they are not. Among 12 countries without recognised subspecialisations, 9 expressed a desire for future recognition. There are large variations between countries regarding the number of officially recognised subspecialities, ranging from 0 to 12.

**Conclusion:**

Based on responses from ESR National Societies Committee members, radiology subspecialties are recognised in 25 countries, while 12 countries do not have formal recognition. The majority of countries without recognised subspecialisations express interest in having them acknowledged in the future.

**Critical relevance statement:**

This follow-up survey among ESR National Societies presents the recognition status of radiology subspecialisations in Europe. Among the 37 responding countries, there are significant variations in the number of officially recognised subspecialties, ranging from 0 to 12.

**Key Points:**

- Among the 37 ESR National Societies responding countries, between 0 and 12 out of 13 radiology subspecialties are recognised.
- No country recognises all subspecialties.
- The majority of countries without recognised subspecialisations would like them to be acknowledged.

## Objectives

The Accreditation Council in Imaging (ACI) is the accreditation body of the European Board of Radiology and provides a one-step service for continuing medical education (CME) accreditation, supporting the European Accreditation Council for Continuing Medical Education in delivering and harmonising the highest level of CME in imaging.

To investigate the latest trends and developments in the radiological community in CME in Europe and beyond, the ACI has performed surveys on an annual basis from 2017 until 2024.

One of the surveys performed among the European Society of Radiology (ESR) membership in 2021 was about “Subspecialisation in radiology in Europe” [1]. The aim of that survey was to obtain information on officially recognised subspecialities, defined as subspecialty diplomas in one of thirteen fields of the Level III European Training Curriculum for Subspecialisation in Radiology [2].

In the survey, we observed heterogeneity in the responses within countries regarding the existence of subspecialties. Therefore, a follow-up survey was developed in close collaboration with the ESR National Societies Committee, and distributed among the 47 members of the ESR National Societies Committee. The aim was to obtain a more accurate overview with regard to all recognised subspecialties in radiology by official bodies in each respective country.

## Methods

The survey was distributed on the 27th of September 2023 among the members of the ESR National Societies Committee (*n* = 47). Societies were asked to submit only one completed survey per country. If respondents were not fully aware of the situation of one particular subspecialisation, they were encouraged to consult experts within their country to ensure that the results were as comprehensive and accurate as possible. Eight reminders were sent, with the final reminder issued on the 18th of July 2024.

The survey included the following questions: (1) Are subspecialties within radiology officially recognised in your country? If yes, which ones? (2) If an area of subspecialisation is not officially recognised in your country, would you like it to be recognised?

In accordance with National Health Service Health Research Authority criteria, this study did not require ethical approval [3].

## Results

### Responses were received from 37 out of 47 countries (78%)

Radiology subspecialties are recognised in 25 countries, while in 12 countries, they are not (Table 1). Among the countries without recognised subspecialisations, the majority (9 out of 12) expressed a desire for subspecialisation recognition in the future (Table 1).

**Table 1:** General recognition of radiology subspecialties in ESR National Societies countries

Country	Are subspecialties within radiology officially recognised in your country?	If an area of subspecialisation is not officially recognised in your country, would you like it to be recognised?
Albania	Yes	Yes
Armenia	Yes	Yes
Belarus	Yes	Yes
Cyprus	Yes	Yes
Finland	Yes	No
France	Yes	No
Germany	Yes	No
Greece	Yes	Yes
Hungary	Yes	Yes
Iceland	Yes	Yes
Israel	Yes	Yes
N. Macedonia	Yes	Yes
Moldova	Yes	Yes
Netherlands	Yes	No
Portugal	Yes	Yes
Romania	Yes	Yes
Russia	Yes	Yes
Serbia	Yes	Yes
Slovakia	Yes	Yes
Slovenia	Yes	Yes
Spain	Yes	Yes
Sweden	Yes	Yes
Switzerland	Yes	Not applicable
Turkey	Yes	Yes
United Kingdom	Yes	No
Austria	No	Yes
Belgium	No	Yes
Estonia	No	Yes
Georgia	No	Yes
Ireland	No	Not applicable
Italy	No	No
Lithuania	No	Yes
Luxembourg	No	Yes
Malta	No	Yes
Norway	No	Not applicable
Poland	No	Yes
Ukraine	No	Yes

Additionally, the responses regarding the desire for subspecialty recognition in countries where they are not yet recognised are also presented

A detailed analysis revealed large variations in the number of officially recognised subspecialities across countries, ranging from 0 to 12. No country recognises all thirteen subspecialties as defined by the Level III ECTSR [2] (Table 2). According to the responses, the highest number of recognised subspecialty diplomas are in Slovenia (*n* = 12), Switzerland (*n* = 11), and Finland (*n* = 10). The most commonly recognised subspecialties across all countries are Interventional Radiology (*n* = 18), Neuroradiology (*n* = 13), and Gastrointestinal and Abdominal Radiology, along with Paediatric Radiology (*n* = 10 each). Medical Imaging Informatics is not recognised as a subspecialty in any country. Several countries provided additional information about the status of subspecialisation within their respective countries in the comment section of the survey. In the Netherlands, Gynaecological and Obstetric, as well as Urogenital Radiology, are integrated in the Gastrointestinal and Abdominal Radiology subspecialisation. Neurointerventional Radiology is recognised as a separate subspecialty in Finland, while Advanced Interventional Radiology holds distinct recognition in France. In France, the official national diploma in Radiology offers access to perform and report all subspecialty exams except Advanced Interventional Radiology. Recognition of expertise in 1 or 2 domains during residency training is possible in France (Table 2). In Greece, the only official subspecialisation is Interventional Radiology. All the remaining domains are covered by radiologists who have a special interest in the topic. In Ireland, the Medical Practitioners Act does not allow the Medical Council to recognise subspeciality status. In Poland, radiology subspecialisation is non-existent either. In Italy, the official law considers three separate specialities, i.e. Diagnostic Radiology, Therapeutic Radiology, and Nuclear Medicine. Neuroradiological expertise and Breast cancer screening programme could be requested only in the form of documented clinical experiences.

**Table 2:** Detailed ESR Level III subspecialties recognition among respondents from ESR National Societies, listed in descending order of recognition

Subspecialty/country	SLO	CH	FI	NL	IL	MD	E	MK	ARM	P	H	SRB	D	IS	RO	S	AL	BY	CY	GR	RUS	SK	TR	UK	F	Total
Interventional radiology	Y	Y	Y	Y	Y		Y	Y		Y	Y	Y		Y	Y			Y	Y	Y	Y	Y		Y		18
Neuroradiology	Y	Y	Y	Y	Y	Y	Y	Y			Y	Y	Y	Y		Y										13
Breast radiology	Y	Y	Y	Y	Y	Y	Y	Y							Y		Y									10
Gastrointestinal and Abdominal Radiology	Y	Y	Y	Y	Y	Y	Y	Y	Y			Y														10
Paediatric radiology	Y	Y	Y	Y	Y	Y	Y				Y		Y										Y			10
Chest radiology	Y	Y	Y	Y	Y		Y	Y	Y	Y																9
Musculoskeletal radiology	Y	Y	Y	Y	Y	Y	Y	Y																		8
Cardiac and vascular radiology	Y	Y	Y	Y	Y				Y	Y																7
Head and neck radiology	Y	Y	Y	Y		Y				Y						Y										7
Emergency radiology	Y	Y	Y				Y																			4
Urogenital radiology	Y	Y				Y		Y																		4
Oncologic imaging	Y					Y			Y																	3
Medical imaging informatics																										
Total	12	11	10	9	8	8	8	7	4	4	3	3	2	2	2	2	1	1	1	1	1	1	1	1	0	
Other (please specify)			*1	*2					*3																*4	

Country codes [8]: *AL* Albania, *ARM* Armenia, *BY* Belarus, *CY* Cyprus, *FI* Finland, *F* France, *D* Germany, *GR* Greece, *H* Hungary, *IS* Iceland, *IL* Israel, *MK* North Macedonia, *MD* Moldova, *NL* Netherlands, *P* Portugal, *RO* Romania, *RUS* Russia, *SRB* Serbia, *SK* Slovakia, *SLO* Slovenia, *E* Spain, *S* Sweden, *CH* Switzerland, *TR* Turkey, *UK* United Kingdom

^*^1 Neurointerventional radiology

^*^2 Gynaecological and obstetrics, and urogenital radiology are integrated in the abdominal subspecialisation

^*^3 Nuclear Medicine is recognised as a separate speciality (not within radiology)

^*^4 Advanced interventional radiology. Recognition of expertise in 1 or 2 domains during residency training is possible

## Discussion

Since the introduction of the first European Specialist Diploma Examination in 1984, more than 30 disciplines now organise European examinations every year under the European Union of Medical Specialists (UEMS)–Council of European Specialist Medical Assessment (CESMA) (UEMS–CESMA) umbrella [4].

Medical subspecialties are also becoming increasingly structured. Therefore, the aim of this study was to provide an additional comprehensive overview of radiology subspecialties in each country, as assessed by ESR National Societies, and make the findings available to the radiology community.

Since the 2023 analysis [1] three new ESR endorsed subspecialty diplomas/certifications have been introduced: the European Diploma in Gastrointestinal and Abdominal Radiology, organized by the European Society of Gastrointestinal and Abdominal Radiology (ESGAR); the European Society of Thoracic Imaging Lung Cancer Screening Certification (ESTI LCS), organized by ESTI [5]; and the European Certification in Prostate MRI, provided by European Society of Urogenital Radiology (ESUR) [6] (Table 3).

**Table 3:** Overview of existing European subspecialty exams and diplomas in radiology and their endorsement

Subspecialty (Level III)	European subspecialty society	European subspecialty exam and diploma	European subspecialty certification	ESR endorsement	UEMS-CESMA appraisal	Title
Breast radiology	EUSOBI	EDBI		Yes		EBBI
Cardiac and vascular radiology	ESCR	EBCR		Yes		EBCR
Chest radiology	ESTI	ESTI diploma		Yes		
	ESTI		ESTI LCS	Yes		
Emergency radiology	ESER	EDER		Yes		FESER
Gastrointestinal and abdominal radiology	ESGAR	Yes		Yes		
Head and neck radiology	ESHNR	EBiHNR diploma		Yes		
Interventional radiology	CIRSE	EBIR		Yes	Yes	EBIR
Musculoskeletal radiology	ESSR	Yes		Yes		EDiMSK
Neuroradiology	ESNR	EBNR (multiple)			Yes	
Oncologic imaging	ESOI	No		–	–	–
Paediatric radiology	ESPR	EDiPR				
Urogenital radiology	ESUR	Yes		Yes		EDiUR
	ESUR		European certification in prostate MRI	Yes		
Medical imaging informatics	EUSoMII	No		–	–	–

List of subspecialties in radiology (according to the European Training Curriculum for Subspecialisation in Radiology), their corresponding European subspecialty society, the name of an existing European Subspecialty Exam and Diploma, endorsement by the ESR and/or the CESMA, and title to be added behind the name of successful candidates indicating full subspecialisation in radiology as allowed by the subspecialty society

*CESMA* Council of European Specialist Medical Assessment, *CIRSE* Cardiovascular and Interventional Radiological Society of Europe, *EBBI* European Board of Breast Imaging, *EBCR* European Board of Cardiovascular Radiology, *EBiHNR* European Board in Head and Neck Radiology, *EBIR* European Board of Interventional Radiology, *EBNR* European Board of Neuroradiology, *EDBI* European Diploma in Breast Imaging, *EDER* European Diploma in Emergency Radiology, *EDiMSK* European Diploma in Musculoskeletal Radiology, *EDiPR* European Diploma in Paediatric Radiology, *EDiUR* European Diploma in Urogenital Radiology, *ESCR* European Society of Cardiovascular Radiology, *ESR* European Society of Radiology, *ESTI* European Society of Thoracic Imaging, *ESTI LCS* European Society of Thoracic Imaging Lung Cancer Screening Certification, *ESER* European Society of Emergency Radiology, *ESGAR* European Society of Gastrointestinal and Abdominal Radiology, *ESHNR* European Society of Head and Neck Radiology, *ESNR* European Society of Neuroradiology, *ESOI* European Society of Oncologic Imaging, *ESPR* European Society of Paediatric Radiology, *ESSR* European Society of Musculoskeletal Radiology, *ESUR* European Society of Urogenital Radiology, *EUSOBI* European Society of Breast Imaging, *EUSoMII* European Society of Medical Imaging Informatics, *FESER* European Fellow of Emergency Radiology, *UEMS* European Union of Medical Specialists

As shown by the answers received, there is a heterogeneity in the countries of the ESR national societies with regard to the existence of officially recognised subspecialities within radiology, ranging from 0 to 12. Some countries are satisfied with their status quo of none or only very few recognised subspecialties in radiology, but the majority of countries would like an area of subspecialisation that is not officially recognised in their country to be recognised in the future. The overall results of the current survey, therefore, indicate that there is a desire for greater subspecialty recognition in radiology, also at the level of ESR National Societies. This aligns with a similar desire, expressed by ESR membership in 2023 [1].

A substantial proportion of countries (12 out of 37) still do not recognise radiology subspecialties. Additionally, no country recognises all thirteen subspecialty diplomas, as Medical Imaging Informatics is not recognised as a subspecialty in any country. The responses also revealed two new subspecialties: Neurointerventional Radiology in Finland and Advanced Interventional Radiology in France. All the above highlights the need for further harmonisation and implementation of subspecialty training in radiology at a European level.

The comments in the survey also identified several disciplines that are not part of the thirteen ESR-recognised subspecialties (Nuclear Medicine, Gynaecological and Obstetric radiology, Breast cancer screening programme, and Lung cancer screening programmes), representing possible further fields of increasingly structured radiology subspecialties or certifications.

As the UEMS–CESMA appraisal for subspecialty exams in radiology is currently limited, an additional challenge for the radiology subspecialty societies, in collaboration with the ESR National Societies Committee, could be to achieve UEMS–CESMA endorsement for more subspecialties. Currently, only the subspeciality diploma offered by the Cardiovascular and Interventional Radiological Society of Europe (CIRSE), the European Board of Interventional Radiology (EBIR) is endorsed by the ESR and the UEMS-CESMA. The European Board of Neuroradiology (EBNR) provides multiple diplomas in the field of neuroradiology [7]. All EBNR diplomas are only endorsed by the UEMS-CESMA.

One of the limitations of this survey is a response rate of only 78% (37 out of 47 ESR National Societies). Therefore, information about the situation of all recognised subspecialties in radiology in the countries of the ten ESR National Societies is still lacking.

Despite increasingly structured subspecialisations, it is still equally important to remember that, in the future, both general radiological knowledge and subspecialised radiological expertise will remain crucial to maintaining a high standard of radiology during all hours of 24/7 service [1].

## Conclusions

According to ESR National Societies, radiology subspecialties are recognised in 25 European countries, while in 12 countries they are not. No country recognises all subspecialties. The majority of countries without recognised subspecialisations would like these areas to be recognised in the future.

## Data Availability

The dataset used and analyses are available from the corresponding author on reasonable request.

## Abbreviations

ACI: Accreditation Council of Imaging
CESMA: Council of European Specialist Medical Assessment
CIRSE: Cardiovascular and Interventional Radiological Society of Europe
CME: Continuing medical education
EBIR: European Board of Interventional Radiology
EBNR: European Board of Neuroradiology
ESGAR: European Society of Gastrointestinal and Abdominal Radiology
ESR: European Society of Radiology
ESTI LCS: European Society of Thoracic Imaging Lung Cancer Screening Certification
ESUR: European Society of Urogenital Radiology
UEMS: European Union of Medical Specialists

## References

[1] Rupreht M, Ricci P, Prosch H, Adriaensen MEAPM (2023) Subspecialisation in radiology in Europe, a survey of the accreditation council of imaging. Insights Imaging 14:159. 10.1186/s13244-023-01481-y 37749296 10.1186/s13244-023-01481-y PMC10519886

[2] ESR (2025) European training curriculum for radiology. Available via https://www.myesr.org/education/training-curricula/. Accessed 17 Mar 2025

[3] NIHRMRC (2025) Is my study research? Decision tool. http://www.hra-decisiontools.org.uk/research/. Accessed 11 Mar 2025

[4] CESMA (2025) Council of European Specialist Medical Assessment. https://www.uems.eu/european-exams. Accessed 17 Mar 2025

[5] https://www.myesr.org/education/. Accessed 17 Mar 2025

[6] European Society of Urogenital Radiology (2025) European certification in prostate MRI. Available via https://www.esur.org/european-certification-in-prostate-mri/. Accessed 23 Mar 2025

[7] EBNR (2025) Excellence in neuroradiology. Available via https://www.ebnr.org/. Accessed 17 Mar 2025

[8] European Commission (2025) Nation codes—digital tachograph. Available via https://dtc.jrc.ec.europa.eu/dtc_nation_codes.php.html. Accessed 19 Mar 2025

